# Supplementary material for: Sleep quality and associated factors among people with asthma at public hospitals in east gojjam zone, North West Ethiopia, 2022
Source: PLoS One. 2025 May 21;20(5):e0323284. doi: 10.1371/journal.pone.0323284 (PMC12094734; doi:10.1371/journal.pone.0323284)
Supplement: Attachment — Submitted filename: response to reviewers (Revised ).docx [file pone.0323284.s004.docx]

To: The Editor, Reviewers, PLOS ONE
Date: December 20, 2023
Subject: Addressing Editor/Reviewer Comments for **the manuscript ‘‘Sleep Quality and Associated Factors among People with Asthma at Public Hospitals in East Gojjam Zone, North West Ethiopia, 2022”** (PONE-**D-23-08092**

Dear Editor, Reviewers,
Many thanks to you for your helpful feedback. We have now tried to address all your concerns to the best of our abilities. Kindly help us with your further concerns (if any) to improvise our paper. Below we are providing our responses to the comments and actions that we took to address them. The responses are highlighted in red.

Thank you. Kind regards,
Authors of PONE-PONE-D-23-08092

# Editor’s Comments

# Comments: Thank you for your nice work. While acknowledging the scientific validity of this study, I would like to kindly request that the authors make significant improvements to the manuscript to meet the quality standards required for the journal. The current version of the manuscript appears to have several shortcomings. I would strongly recommend that the authors seek support form a native English speaker for thorough editing. There are instances of spelling errors and incomplete sentences (grammatically incorrect) and formatting issues (e.g., table presentation) observed throughout the manuscript. I would suggest your attention to these details may greatly enhance the overall quality of the work.

# Response: Thank you, dear editor, now we have tried to fix those problems.

# All the revisions that we made are highlighted in red in our Tracked Changes version of the manuscript

# Authors also need to attend the following major points in addition to the reviewers' comments. Comment: The sample size determination was based on a prevalence rate from a study conducted in Nigeria. To what extent can the prevalence of asthma and related factors be considered similar between Nigeria and East Gojjam Zone in Ethiopia, and how might this affect the generalizability of the findings? How might the regional and cultural differences between Nigeria and East Gojjam Zone affect the generalizability of the findings? I couldn’t find something related to this in the manuscript.

# Response: Thank you dear editor for pointing out these important concerns. Initially as there were no previous researches conducted in Ethiopia, we had intended to utilize a 50% prevalence rate. However, after some discussion, we concluded that, given that both Ethiopia and Nigeria are in Africa, there might not be many notable regional and cultural differences between them and the generalizability of the findings may not be greatly affected. That was why we used the prevalence rate from the study conducted in Nigeria.

# Comment: How did authors manage the potential sources of bias in data collection, given that the data was collected by trained nurses who were also working in the chronic disease follow-up outpatient department? Have you considered how this affect the accuracy of the results might?

# Response: Thank you dear editor for showing us this critical mistake, this was typing error, we wanted to say who were working out of the chronic disease follow up outpatient department

# Page 11 line # 234 methods part of the manuscript

# Comment: The study considers various factors, including clinical, psychosocial, and behavioural variables, as potential predictors of poor sleep quality among people with asthma. Could you elaborate on the theoretical basis or prior research that supports the inclusion of these specific variables in the analysis?

Response: Thank you dear editor, as we tried to address in the introduction part of the manuscript, there were prior studies that showed different factors for poor sleep quality among people with asthma, such as clinical-related factors (severe asthma, uncontrolled asthma, and a longer medical history) and behavioral- and psychosocial-related factors (poor sleep hygiene practices, smoking, poor social support, stress, and depression).

# Page 5 line # 95-97 in the introduction part of the manuscript

**Comment**: This study was based on self-reported data for variables such as sleep quality, social support, and substance use. How might self-reporting introduce biases and limitations in the study's results, and what steps were taken to mitigate these potential issues?

**Response:** Thank you, dear reviewer, for pointing out these important issues. Since the data for the variables were collected through face-to-face interviews, participants might not remember past events accurately, and they might provide socially acceptable responses rather than truthful ones (i.e., recall bias and social desirability bias might be introduced during data collection). To reduce these issues, the data collectors describe the objectives of the interview and gave the participants appropriate time and selected a quiet area during the interview to reduce destruction so that they felt comfortable, freely remembered the events, and, as much as possible, tried to give the correct response to the questions. In addition, to reduce social desirability bias, the data collectors gave strong awareness to the participants that their privacy and confidentiality would be ensured and the information they gave would remain private, so that they were more likely to answer sensitive questions openly and correctly.

**Comment**: Different data ascertainment tools were used to assess several factors. How does the reliability and validity of these assessment tools impact the robustness of your study’s findings, and were these tools validated for the specific population studied in East Gojjam Zone in Ethiopia?

As we tried to describe in the methods section of the manuscript, the tools which were used for assessing the variables in our study were standardize, valid and reliable and different studies in Ethiopia were conducted using these tools.
**Comment**: Would you please provide evidence why did you use P-value of <0.25 to include variables in multivariable logistic regression (at least provide citations)?

**Response:** Thank you, dear reviewer, for pointing out these important issues. As many researchers agree, p-values less than 0.2 and 0.25 are mostly used for screening variables included in multivariable logistic regression. We specifically used a p-value <0.25 in order to include important variables that might be missed if we used a p-value <0.2 as a cut point and the model may be over-fitted if we use the higher level ( p-value >0.25) by including variables that are less importance (Greenland, 1989).

**Comment**: The discussion section lacks a critical element. In addition to comparing your study with similar research, the authors should also delve into the significance of their findings within the context of Ethiopian or East African or African literature. Furthermore, it is essential for the authors to explore the policy, practice, and research implications arising from their findings.

Response: Thank you, dear reviewer, for your suggestion. Now the significance of our study findings is added in the discussion section of the manuscript as per suggestion.

# Page 22 line # 392-398 in the discussion part of the manuscript

**Reviewers' comments:**

**Reviewer #1: comments**

**Comment**: Over all, the manuscript should be edited for English language and grammar issues.

Response: Thank you, dear reviewer, for your constructive comments. Now we have tried to fix the problems and thoroughly checked our text for grammatical inconsistencies

# All the revisions that we made are highlighted in red in our Tracked Changes version of the manuscript

**Comment:** **Abstract:** "The magnitude of poor sleep quality among people with... is widespread and has detrimental consequences "This sentence is not full; it lacks words.

**Response:** Thank you dear reviewer, now we have fixed the problem by adding the word asthma

# Page 2 line # 23 in the abstract part of the manuscript

# Comment: It is better to include the gap of this study in the background part of the abstract.

Response: Thank you, dear reviewer for your suggestion, now we have tried to show the gap of this study and kindly include the following text in the abstract part: However, it has not been well studied especially in low income countries including Ethiopia.

# Page 2 line # 26-27 in abstract part of the manuscript

**Introduction
Comment:** It is better to show in detail the pathophysiology of sleep and asthma.

**Response:** Thank you, dear reviewer for your suggestion. Now we tried to add the pathophysiology of sleep and asthma in the introduction section of the manuscript as per suggestion.

# Page 4 line # 82-84 in the introduction part of the manuscript

**Comment:** I think, despite it not being done in the study area, there are studies that show related magnitude with almost related factors. What different considerations or approaches did researchers include for the current study? What is the difference between this study's different outputs of sleep quality and those of other medical illnesses, even those done in Ethiopia?
**Response:** Thank you, dear reviewer. In Ethiopia, some studies were conducted regarding sleep quality and associated factors among people with different chronic illnesses but investigations on this issue in people with asthma have not been done. Most of these studies were conducted only at a single hospital and the researchers missed one important variable (sleep hygiene practices), which greatly affect the sleep quality of the individuals. But our study included eleven hospitals, and sleep hygiene practices was also assessed.

**Methods
Comment:** what is the reason behind using non-probability sampling? Is there any scientific reason to exclude participants with a follow-up of less than 2 months? I think it was also possible to take all 576 participants without considering allocation. What was the reason to use backward variable selection rather than the defaults enter method?

**Response:** Thank you, dear reviewer, for pointing out these important issues. The reason that we excluded participants with a follow-up of less than 2 months was because, based on the Global Initiative for Asthma (GINA) 2021 management protocol, asthma severity is assessed retrospectively after at least 2-3 months of treatment at the level of treatment required to control symptoms and exacerbations.

We used the non-probability sampling technique because there were no sampling frames and the source populations were small, so we could not use simple random sampling techniques as well as systematic sampling techniques.

We preferred using the backward variable selection process because this method starts with a full model that considers all of the variables to be included in the model, and the least significant variable is deleted first, removing those that are irrelevant or redundant, and including a set of variables that will provide the best fit for the model so that accurate predictions can be made. It has the advantage of assessing the joint predictive ability of variables, as the process starts with all variables being included in the model, removes the least important variables early, and leaves only the most important variables in the model.

**Result
Comment:** Please limit the number of figures; it is not important to describe age separately by figure; rather, you can use sociodemographic tables. Similarly, for controlled asthma and the like, 18.2% of participants cannot read and write. Have you approached those regarding ethical issues for such people? Since nothing is reported for them.

**Response:** Thank you, dear reviewer, for your suggestion. Now the numbers of figures are limited to 2, age category is added in sociodemographic table and level of asthma control is added in clinical related variables table. Since the data were collected face-to-face, nothing was done for the participants who could not read or write except the data collector repeatedly reading the question until the participants understood it.

- Age category is added in table 1 and asthma control is added in table 2 page 14 and 15 respectively ( highlighted in red) in the result part of manuscript

**Comment:** From your findings, 64.3% of respondents use alcohol is currently. Which type of alcohol, and how could participants reported, specifies this question? I consider that almost all of the people taking medication for asthma are recommended to stop drinking, and your participants are also those with follow-ups lasting more than 2 months. How could 64.3% of participants use alcohol?

**Response:** Thank you, dear reviewer, for pointing out these important issues. Current substance use was assessed using the Alcohol, Smoking, and Substance Involvement Screening Test (ASSIST). Based on this tool, the participants were asked whether they had used alcohol (any type), cigarettes, or chat for the last three months. If their response was yes for any of the listed substances, they were considered a current substance user. So, current alcohol use was assessed in this way.

Of course, all the participants were taking medications; if so, alcohol consumption is not recommended. But, as you see, more than half of the participants were current alcohol users. Even if it needs further investigation, this might be due to a lack of awareness or information on stopping alcohol consumption while they are taking medications.

**Comment:** It is better to include all variables associated with bivariate logistic regression within the regression table rather than only those associated with multivariable analysis.

**Response:** Thank you, dear reviewer for your suggestion. Now all the variables associated with bivariate logistic regression are added in regression table

# Page 21 table 5 highlighted in red in the result part of the manuscript

**Discussion
Comment:** The possible difference in findings should be well explained using scientific evidence, specifically how sample size and cultural variation make variation within the indicated countries, etc.

**Response:** Thank you, dear reviewer for your constructive suggestion. Now we have tried to explain the possible difference between our study findings and those of other studies using scientific evidence (we have cited them).

- We have added reference # 9, 42, 43 & 44 at page 22 and 23 in the manuscript

**Comment:** Limitation, please specify recall bias? For which data? How social desirability could be a limitation for this study?

**Response:** Thank you, dear reviewer, for raising these important points. Recall bias might be a limitation for the data on the variables collected through self-report, including sleep quality, level of asthma control, social support, sleep hygiene practices, anxiety, stress, and current substance use, since they need to remember to answer specific questions used to assess each variable.

The participant might give answers that are more socially acceptable than their true opinions or behaviors. For example, someone might feel obligated to lie about habits like smoking or drinking alcohol which can lead to distorted conclusions about the study.

Considering these issues we tried to minimize their effects as we describe it in editor’s comment number 4

**Comment:** Your recommendation to the researcher: what is the reason for your being unable to do a follow-up study?

**Response:** Thank you, dear reviewer; we were unable to conduct a follow-up study because of shortage of time.

**Reviewer #2: Comments**
**Comment:** thank you for coming up with this important topic. Overall, your study provides good insights into the sleep quality issues of people with Asthma.

**Response:** Thank you, dear reviewer for your encouragement
**Abstract
Comment:** Page 2, line 23, you want to say with Asthma.

**Response:** Thank you, dear reviewer for correcting us. Now the word asthma is added

- Page 2 line 23 in abstract part of the manuscript

**Comment:** It would be worth it if you stated the gaps in the literature and then the aim in the introduction.
Recommendation: do you identify any gaps in the advice? a recommendation should be based on your findings. Should is not a good term for recommendations.
**Response:** Thank you, dear Reviewer for your constructive suggestions, now we have tried to address these issues as per suggestion.

- Page 25 line 461-467 in the conclusion and recommendation section and page 3 line 45-47 in the abstract section of the manuscript

**Introduction**
**Comment:** The Introduction is good. But it can be excellent if you rewrite it to
For example, you can rewrite the first sentence like this:” Asthma is a chronic lung disease characterised by airway hyperresponsiveness to various direct and indirect stimuli, leading to variable symptoms of wheeze,,,,,,,,,,,,. Globally, it affects --- of individuals, accounting -% of individuals and responsible for, death …
Sleep is a crucial human physiological process important for the proper functioning of the brain and the overall well-being of the human body. It accounts for approximately an individual's life.
I would rewrite the whole introduction so that all the sentence structure and grammatical and punctuation errors will be resolved.
**Response:** Thank you, dear reviewer for giving us clear directions on how to fix the problems in the introduction part of the manuscript. Now we have tried to re write the introduction part as per your suggestions.

- Page 4 line 68-70 and line 75-77 in the introduction part of the manuscript

**Method**

**Comment:**
The method part is very excellent and detailed, but it lacks synthesis.
-Delete and between MLPH & LPH
-I would detail the hospital settings, how many people they are serving, their care about Asthma
I would restructure the methods like this.
1. Study design and setting
2. Population/Study participants and sampling/ sample size estimations.
3. Study variables: you can state the variables and their definition simultaneously.
4. Data collection
5. Statistical analysis
**Response:** Thank you, dear Reviewer, now we have tried to address these issues as per suggestion.

- Page 6 line 118-124 and line 130 in the methods part of the manuscript

**Result**

**Comment:** The results were well presented.

**Response:** Thank you, dear reviewer, for your encouraging comments

**Discussion
Comment:** I suggest adding a paragraph or two on the implications of your study in the discussion.
**Response:** Thank you, dear reviewer, now we have added the implications of our study findings in the discussion section of the manuscript.

- Page 22 line 392-398 in the discussion part of the manuscript

**Limitation
Comment:** How do you handle limitations such as recall and social desirability biases?

**Response:** Thank you, dear reviewer for asking us to clarify these important issues. To reduce these issues, the data collectors gave the participants appropriate time and selected a quiet area during the interview to reduce destruction so that they felt comfortable, freely remembered the events, and, as much as possible, tried to give the correct response to the questions. In addition, to reduce social desirability bias, the data collectors gave strong awareness to the participants that their privacy and confidentiality would be ensured and the information they gave would remain private, so that they were more likely to answer sensitive questions openly and correctly.

**Conclusion**
**Comment:** I would suggest saying “Conclusion and recommendation” or conclusion and stating the pertinent recommendations rather than having a separate section for recommendations.

**Response:** Thank you, dear reviewer for your suggestion. We have corrected it as per suggestion

- Page 25 line 456 in the manuscript

References
