# Supplementary material for: Sleep quality and associated factors among people with asthma at public hospitals in east gojjam zone, North West Ethiopia, 2022
Source: PLoS One. 2025 May 21;20(5):e0323284. doi: 10.1371/journal.pone.0323284 (PMC12094734; doi:10.1371/journal.pone.0323284)
Supplement: Attachment — Submitted filename: Reveiwer 1.docx [file pone.0323284.s003.docx]

**Reviewer`s comment**

1. 79-81: it is too long sentence. The concept^referenced from articles, its meaning is distorted. Please, reconstruct it.
2. 88: the concept you have mentioned here is not there in your reference

- (Malek F, Sayah SK, Kia NS, Ghods E. The Relationship Between Sleep Quality and Quality of Life Among Patients With Asthma. Cureus. 2022;14(3).)
- The page number :14 you wrote is not there on the refference.

3. 90-91: “have poor academic Performance” this is not the issue related with asthma (I mean with your title). check you reference (9)(18)(19)(21). The reference you need to use here, must match your research title.
4. 106-107: I think there is a study conducted in Ethiopia. “Prevalence and determinants of insomnia among patients living with asthma in Northwest Ethiopian University specialised hospitals: Multicentre cross-sectional study” brows it.
5. 149: “576 people with asthma had follow-up in the hospitals” source of this information must be known and cited.
6. 237: Data collection procedure was not discussed.Please, address how did you make contact with your respondents. From which client you had started? How the procedure was looks like.
7. 271: “response rate of 100%” Since these people are asthmatic patients, how did you get the respondents free from asthmatic attack during data collection? Even if they were clients attending follow-up clinics, this could not make them free from attack. What was your action when you found someone in respiratory distress? Since 35.2% of your respondents were suffering from severe asthma, So how did you handle the situation. Your report seems somewhat illogical. You have mentioned in your study the severity of the client's condition.

427-428: “because individuals with very poorly controlled asthma have daily symptoms of asthma such as coughing, chest tightness, and shortness of breath and may experience nighttime flare-ups,”

8. 292:your table missed “sex of respondents”. why? Since it was one of your independent variable. Why you remove from the entire study?
9. 310: (57.1%) of the participants took 16–30 minutes to fall asleep (Figure 1, Figure 2). I didn’t get this information on the mentioned area.
10. 389-390: “no research had been conducted in Ethiopia.” please check the 4^th^ comment.
11. 392-398: just discus the result.
12. 415: “study conducted in the USA might be due to the difference in the study design.” Your study design is cross-sectional, so the recommended reference for discussion should be a similar study design.
13. 417: “fact that the participants in the study conducted in Brazil were only females (9, 44).” You cannot use this reference for comparison because they are studies with different populations.
14. 457:” The magnitude of poor sleep quality among people with asthma was high in this study” why you need to conduct this study? It is obvious that people with asthma, definitely experiencing poor sleep quality.
15. (31) Bøen H, Dalgard OS, Bjertness E. The importance of social support in the associations between psychological distress and somatic health problems and socio-economic factors among older adults living at home: a cross sectional study. BMC geriatrics. 2012;12(The page number you have mentioned is not correctly mentioned)
16. Generally, fixing the reference you had used for its similarity with you title.
