# Supplementary material for: Sleep quality and associated factors among people with asthma at public hospitals in east gojjam zone, North West Ethiopia, 2022
Source: PLoS One. 2025 May 21;20(5):e0323284. doi: 10.1371/journal.pone.0323284 (PMC12094734; doi:10.1371/journal.pone.0323284)
Supplement: Attachment — Submitted filename: Response to reviewers.docx [file pone.0323284.s005.docx]

To: The Editor, Reviewers, PLOS ONE
Date: October 30, 2024
Subject: Addressing Editor/Reviewer Comments for  **the manuscript ‘‘Sleep Quality and Associated Factors among People with Asthma at Public Hospitals in East Gojjam Zone, North West Ethiopia, 2022”**  (PONE-**D-23-08092R1)**

Dear Editor, Reviewers,
Many thanks to you for your helpful feedback. We have now tried to address all your concerns to the best of our abilities. Kindly help us with your further concerns (if any) to improvise our paper. Below we are providing our responses to the comments and actions that we took to address them. The responses are highlighted in red.

Thank you. Kind regards,
Authors of PONE-PONE-D-23-08092R1

**Reviewers' comments:**

**Reviewer #1: comments**

**Comment**: 79-81: it is too long sentence. The concept^referenced from articles, its meaning is distorted. Please, reconstruct it.

**Response:** Thank you dear reviewer, now we have fixed the problem and we tried to rewrite it as per your suggestion.

- Page 4 line # 79-82

**Comment:** 88: the concept you have mentioned here is not there in your reference

- (Malek F, Sayah SK, Kia NS, Ghods E. The Relationship Between Sleep Quality and Quality of Life Among Patients With Asthma. Cureus. 2022;14(3).)
- The page number :14 you wrote is not there on the refference.

**Response:** Thank you dear reviewer for your constructive comments. We have now included the correct information which was found in the reference and the problem is fixed.

- Page 5 line # 89-90

Comment: 90-91: “have poor academic Performance” this is not the issue related with asthma (I mean with your title). Check you reference (9)(18)(19)(21). The reference you need to use here, must match your research title.

# Response: Thank you, dear reviewer for your constructive comments and suggestions. Now we have removed the references which were unrelated to our issues.

**Comment:** 106-107: I think there is a study conducted in Ethiopia. “Prevalence and determinants of insomnia among patients living with asthma in Northwest Ethiopian University specialised hospitals: Multicentre cross-sectional study” brows it.

**Response:** Thank you, dear reviewer for your suggestion. We have searched for and accessed it. From the beginning, this study (“Prevalence and Determinants of Insomnia among Patients Living with Asthma in Northwest Ethiopian University Specialized Hospitals”) was published in 2023, after we conducted our study. Even, it had been published before our study, the two studies are different. That is why we stated that no similar studies have been conducted in Ethiopia.

**Comment:** 149: “576 people with asthma had follow-up in the hospitals” source of this information must be known and cited.

**Response:** Thank you, dear reviewer constructive comment and suggestion. We found the information from the chronic follow up OPD registration book from each hospital.

**Comment:** 237: Data collection procedure was not discussed. Please, address how did you make contact with your respondents. From which client you had started? How the procedure was looks like.

**Response:** Thank you, dear reviewer, for pointing out these important issues. Now we have tried to address these issues as per your suggestion.

- Page 11 line # 235-239 highlighted in the method part of the manuscript

**Comment:** 271: “response rate of 100%” Since these people are asthmatic patients, how did you get the respondents free from asthmatic attack during data collection? Even if they were clients attending follow-up clinics, this could not make them free from attack. What was your action when you found someone in respiratory distress? Since 35.2% of your respondents were suffering from severe asthma, So how did you handle the situation. Your report seems somewhat illogical. You have mentioned in your study the severity of the client's condition.

427-428: “because individuals with very poorly controlled asthma have daily symptoms of asthma such as coughing, chest tightness, and shortness of breath and may experience nighttime flare-ups,”

**Response:** Thank you, dear reviewer, for raising these important issues. There were no clients with asthmatic attack at chronic follow-up OPD, because clients having acute asthmatic attacks were evaluated and treated at emergency. So that, clients who were available at follow-up OPD during data collection period were stable, and the data collectors did not face difficulties during the interview. None of the respon dents terminated the interview.

Additionally, clients with severe asthma do not necessarily mean they are experiencing acute attack or respiratory distress. As defined, severe asthma is a condition requiring GINA treatment Step 4/5 (moderate or high dose inhaled corticosteroid/ long acting beta agonist (ICS/LABA) plus add-on therapy) to be controlled. Therefore, 35.2% of the respondents were individuals on GINA treatment step 4/5 or who used moderate or high dose inhaled corticosteroid/ long acting beta agonist (ICS/LABA) plus add-on therapy) to control their asthma symptoms rather than those experiencing asthmatic attack or respiratory distress. Similarly, individuals with very poorly controlled asthma do not necessarily mean that, their symptoms persist throughout the day rather; it means that, their symptoms may occur daily. However with medications which provide immediate relief of asthma symptoms and long term controller medications, these individuals become stable during their follow up appointment and during the interview.

In fact, the data collectors did not find any participants who were in asthmatic attack. If the data collectors had found participants experiencing asthmatic attack, they would have been advised to take appropriate measures based on the participants’ condition. That is why we achieved 100% response rate.

**Comment:** 292: your table missed “sex of respondents”. Why? Since it was one of your independent variable. Why you remove from the entire study?

**Response:** Thank you, dear reviewer, for pointing out this important issue. We missed this variable in the table, considering that, since it has two categories, it might be sufficient to describe in the text portion. But now we have included it in the table.

- Highlighted in red at page 14 in table 1in the result part of the manuscript.

**Comment:** 310: (57.1%) of the participants took 16–30 minutes to fall asleep (Figure 1, Figure 2). I didn’t get this information on the mentioned area.

**Response:** Thank you, dear reviewer for your suggestion. This information is found at figure 2. Now we have put figure 1 and 2 separately at the text portion of the manuscript for each sentence. Figure one describes about subjective sleep quality and figure 2 is about sleep latency.

**Comment:** 389-390: “no research had been conducted in Ethiopia.” please check the 4^th^ comment

**Response:** Thank you, dear reviewer for your suggestion. Now we have tried to address it at your 4^th^ comment

**Comment:** 392-398: just discus the result.

**Response:** Thank you, dear reviewer, for your suggestion. Now we have tried to modify the issue.

**Comment:** 415: “study conducted in the USA might be due to the difference in the study design.” Your study design is cross-sectional, so the recommended reference for discussion should be a similar study design.

**Response:** Thank you, dear reviewer for these constructive comments. We have now fixed the issues.

**Comment:** 417: “fact that the participants in the study conducted in Brazil were only females (9, 44).” You cannot use this reference for comparison because they are studies with different populations.

**Response:** Thank you, dear reviewer for your suggestion. We have now fixed the issue.

**Comment:** 457:” The magnitude of poor sleep quality among people with asthma was high in this study” why you need to conduct this study? It is obvious that people with asthma, definitely experiencing poor sleep quality.

**Response:** Thank you, dear reviewer for rising this important point. Even though it is commonly said that asthmatic individuals have sleep problems, there were no scientifically documented evidences on the magnitude of poor sleep quality and related factors on those individuals in our country. As health professionals, we should improve the health of asthmatic individuals by enhancing the treatment options, including addressing problems associated with poor sleep. To achieve this, we must scientifically show the magnitude of the problem and its associated factors to the responsible body (policy makers, those who participate in treatment guideline revision…). That is why we conducted this study.

**Comment:** (31) Bøen H, Dalgard OS, Bjertness E. The importance of social support in the associations between psychological distress and somatic health problems and socio-economic factors among older adults living at home: a cross sectional study. BMC geriatrics. 2012;12(The page number you have mentioned is not correctly mentioned).

**Response:** Thank you, dear Reviewer for your constructive suggestions. Now we have tried to resolve the issue as per suggestion.

**Comment:** Generally, fixing the reference you had used for its similarity with you title.

**Response:** Thank you, dear reviewer for providing these constructive comments and suggestions. Now we have tried to correct the issues as per your suggestions.

**Reviewer #4 comments**

**Comment:** One of the shortcomings of this study is the method of data collection, which is the interview. First, explain who conducted the interview and then write the profile and background of the interviewer.

Response: Thank you dear reviewer, for your constructive comments. The data collectors were BSc nurses. The supervisors were also nurses with BSc degrees and above. For instance the supervisors at Yejubie Primary Hospital, Debreelias Primary Hospital, and Debrework primary hospital were BSc nurses, whereas at the other hospitals, the supervisors were MSc nurses.

- We tried to describe this at page 11 line # 233-235 in the method part of the manuscript.

**Comment:** Creating recall bias in this study: Data was collected using face-to-face interviews where participants had to recall their sleep patterns and asthma symptoms, which opens the study to recall bias. Participants may not accurately remember details; especially concerning sleep quality and other self-reported behaviors like alcohol consumption (What measures have you taken to prevent this type of bias?)Creating social desirability bias in this study: Some participants may have underreported behaviors like alcohol use or overstated their sleep quality due to the social stigma around admitting poor health habits or conditions during face-to-face interviews (What measures have you taken to prevent this type of bias?)

**Response:** Thank you, dear reviewer, for pointing out these important issues. Since the data for the variables were collected through face-to-face interviews, participants might not remember past events accurately, and they might provide socially acceptable responses rather than truthful ones (i.e., recall bias and social desirability bias might be introduced during data collection). To reduce these issues, the data collectors describe the objectives of the interview and gave the participants appropriate time and selected a quiet area during the interview to reduce destruction so that they felt comfortable, freely remembered the events, and, as much as possible, tried to give the correct response to the questions. In addition, to reduce social desirability bias, the data collectors gave strong awareness to the participants that their privacy and confidentiality would be ensured and the information they gave would remain private, so that they were more likely to answer sensitive questions openly and correctly.

**Comment:** result: The results were well presented.

Response: thank you dear reviewer for your encouragement.
**Comment:** Discussion: In the discussion section, write a paragraph about the application of the findings in clinical settings.

**Response:** Thank you, dear reviewer, now we have added a paragraph about the applications of our study findings in the clinical settings the discussion section of the manuscript.

- Page 22 line 392-396 in the discussion part of the manuscript
