# Supplementary material for: Sleep quality and associated factors among people with asthma at public hospitals in east gojjam zone, North West Ethiopia, 2022
Source: PLoS One. 2025 May 21;20(5):e0323284. doi: 10.1371/journal.pone.0323284 (PMC12094734; doi:10.1371/journal.pone.0323284)
Supplement: Attachment — Submitted filename: Response_to_reviewers_auresp_3.docx [file pone.0323284.s006.docx]

To: The Editor, Reviewers, PLOS ONE
Date: January 05, 2025
Subject: Addressing Editor/Reviewer Comments for  **the manuscript ‘‘Sleep Quality and Associated Factors among People with Asthma at Public Hospitals in East Gojjam Zone, North West Ethiopia, 2022”**  (PONE-**D-23-08092R2)**

Dear Editor, Reviewers
Many thanks to you for your helpful feedback. We have now tried to address all your concerns to the best of our abilities. Kindly help us with your further concerns (if any) to improvise our paper. Below we are providing our responses to the comments and actions that we took to address them. The responses are highlighted in red.

Thank you. Kind regards,
Authors of PONE-PONE-D-23-08092R2

**Reviewers' comments:**

**Reviewer #5 comments**

**Comment 1:** Poor sleep quality is well known among asthmatic patients and it is worst in the poorly controlled asthma group due to the frequent nocturnal symptoms that the patients have. The prevalence of poor sleep quality in the paper was acquired by subjective feeling of the patients (self-rated -figure 1). I can't find the prevalence based on the PSQI scorer the overall prevalence of poor sleep quality in the study population in this paper.

Response: Thank you dear reviewer, for your constructive comments. Figure 1 illustrates the subjective ratings of the participants' sleep quality, rather than the overall sleep quality as you mentioned in your comment. We previously described the overall prevalence of poor sleep quality based on the PSQI score in text form (line# 308 page 107). We have now included this information in Table 4 on page 18 of the manuscript (highlighted in red in the table).

**Comment 2:** This study should also exclude OSA patients and patients with GERD symptoms which are known to have sleep disturbances.

Response: Thank you dear reviewer, for your constructive comments and suggestions. In the proposal phase of this study, we indicated that participants with obstructive sleep apnea (OSA) and gastroesophageal reflux disease (GERD) would be excluded from the study. However, after data collection, we did not find any participants with these conditions. That is why we did not include this information in the exclusion criteria in the report of this study's results.
