# Supplementary material for: Sleep quality and associated factors among people with asthma at public hospitals in east gojjam zone, North West Ethiopia, 2022
Source: PLoS One. 2025 May 21;20(5):e0323284. doi: 10.1371/journal.pone.0323284 (PMC12094734; doi:10.1371/journal.pone.0323284)
Supplement: Attachment — Submitted filename: Response_to_reviewers_auresp_4.docx [file pone.0323284.s007.docx]

To: The Editor, Reviewers, PLOS ONE
Date: March 24, 2025
Subject: Addressing Editor/Reviewer Comments for  **the manuscript ‘‘Sleep Quality and Associated Factors among People with Asthma at Public Hospitals in East Gojjam Zone, North West Ethiopia, 2022”**  (PONE-**D-23-08092R3)**

Dear Editor, Reviewers
Many thanks to you for your helpful feedback. We have now tried to address all your concerns to the best of our abilities. Kindly help us with your further concerns (if any) to improvise our paper. Below we are providing our responses to the comments and actions that we took to address them. The responses are highlighted in red.

Thank you. Kind regards,
Authors of PONE-PONE-D-23-08092R3

**Journal Requirements:**

Please review your reference list to ensure that it is complete and correct. If you have cited papers that have been retracted, please include the rationale for doing so in the manuscript text, or remove these references and replace them with relevant current references. Any changes to the reference list should be mentioned in the rebuttal letter that accompanies your revised manuscript. If you need to cite a retracted article, indicate the article’s retracted status in the References list and also include a citation and full reference for the retraction notice.

Response: Thank you Dear Editor for your feedback regarding our reference list. As requested, we have carefully reviewed all cited references to ensure accuracy and completeness. We have also conducted a thorough check for any retracted articles and confirm that none of the references included in our manuscript have been retracted. Since no retracted papers are cited, no changes have been made to our reference list.
